# Aquatic vegetation promotes prey diversity but not the diversification of predator phenotypes

**DOI:** 10.1098/rspb.2026.1458

**Published:** 2026-10-14

**Authors:** Rebecca J. Best, Marek Svitok, Moritz D. Lürig, Cameron M. Hudson, Vladimíra D. Carreira, Milan Novikmec, Miguel C. Leal, Ole Seehausen, Philine G. D. Feulner, Blake Matthews

**Affiliations:** ^1^ School of Earth & Sustainability, Northern Arizona University, Flagstaff, AZ 86011, USA; ^2^ Department of Biology and General Ecology, Faculty of Ecology and Environmental Sciences, Technical University in Zvolen, Zvolen 960 01, Slovakia; ^3^ Department of Forest Ecology, Faculty of Forestry and Wood Sciences, Czech University of Life Sciences, Prague, Praha-Suchdol 165 00, Czechia; ^4^ Plant Science and Biodiversity Centre, Institute of Botany, Slovak Academy of Sciences, Bratislava 845 23, Slovakia; ^5^ Department of Fish Ecology and Evolution, Eawag Swiss Federal Institute of Aquatic Science and Technology, Kastanienbaum 6047, Switzerland; ^6^ Florida Museum of Natural History, Gainesville, FL 32611, USA; ^7^ Centre for Ecology, Evolution, and Environmental Changes, Universidade de Lisboa, Lisbon 1749-016, Portugal; ^8^ Centre for Environmental and Marine Studies, University of Aveiro, Aveiro 3810-193, Portugal; ^9^ Institute of Ecology and Evolution, University of Bern, Bern, BE 3012, Switzerland

**Keywords:** adaptive divergence, macrophytes, competition, diet, freshwater fish, invertebrates

## Abstract

Freshwater fish have evolved to forage in a range of habitats, from open water in lakes to benthic shorelines and streams. Much research has focused on how prey differences, such as zooplankton versus benthic macroinvertebrates, might select for divergence in predator traits across this habitat gradient. However, major structural components of this habitat contrast, such as aquatic vegetation, may fundamentally constrain foraging success. Here, we used a mesocosm experiment to test for interactive effects of vegetated habitat and fish density on the survival and growth of phenotypically variable juvenile sticklebacks. We investigated two scales of phenotypic variation: distinct lake and stream ecotypes and F2 lake-stream hybrids. We found that macrophytes strongly increased invertebrate biomass and functional richness, which increased average fish condition. Surprisingly, the relative performance of contrasting ecotypes and genotypes depended only on fish density. Stream ecotypes had higher survival than lake ecotypes, as well as higher condition. In parallel, F2 hybrids had higher growth when homozygous for stream alleles at three quantitative trait loci. Together, these findings suggest that adaptive divergence among habitats depends on the ways that prey type, habitat structure, and genetic variation for feeding and growth traits combine with competition to limit the abundance of accessible prey.

## 1. Introduction

The evolution of foraging efficiency plays an important role in the adaptive divergence of consumers, with well-known examples in fish, birds and mammals [1–3]. However, it is still unclear how forces limiting both the functional composition and accessibility of prey work to promote or constrain character divergence. Whereas much attention has been paid to the relative abundance and nutritional value of alternative prey species [4,5], alternate prey often exist in alternate habitat types, and the costs and benefits of exploiting those prey may depend on a predator's ability to locate and capture them amid changing structural complexity [6,7]. For example, research in a range of aquatic systems has shown that branching vegetation can increase macroinvertebrate abundance and diversity [8,9], but whether this promotes or constrains niche divergence in predators will depend on how habitat context modifies the outcomes of competition for food.

Many of the clearest examples of character divergence in predators come from freshwater fish species adapting to divergent environments, including guppies [10], alewives [11], char [12], whitefish [13] and sticklebacks [14,15]. Evidence for divergence often includes laboratory-assay findings of differential capture efficiency for limnetic versus benthic ecotypes on open water zooplankton versus common benthic invertebrates [16–18]. While valuable, such experiments greatly simplify both prey composition and habitat context. In natural settings, benthic vegetation can modify not only the dominant type of food but also the complexity and accessibility of the food web [19]. By providing both increased surface area for epiphyton [20] and a diversity of hiding spaces, macrophytes alter secondary productivity of herbivorous invertebrates as well as necessary handling time and profitability of prey for predatory insects and fish [21,22].

Importantly, macrophyte presence may also interact with competition to shape selection on feeding strategies. Habitat structure can enhance predator growth rates by increasing the quality and diversity of their prey, or suppress predator performance by decreasing foraging success [21]. Fish with traits suited to capture prey in vegetated areas are more likely to use those areas for foraging [23], but a high density of competitors in these high-quality habitats can lead fish to forage in bare areas instead [24]. At the same time, high densities of competing fish can strengthen selection for divergent diets and foraging strategies [25,26], or can decrease the scope for diet partitioning by entirely depleting one type of food [11]. Thus, the potential for divergent selection on feeding strategy may depend on relative rates of habitat-dependent prey production and density-dependent prey depletion. Despite these potentially strong and interactive effects of physical habitat structure and competition on diet selection, few studies have tested how habitat complexity affects variation in performance among competing and diverging predator phenotypes.

Across the Northern Hemisphere, threespine sticklebacks have repeatedly differentiated into pelagic versus benthic forms in a range of freshwater habitats [27]. Ecotype differentiation in sticklebacks is facilitated by high standing genetic variation in marine source populations, which provides a template for rapid divergence in allele frequencies across both limnetic versus benthic habitats within lakes [27], and lake versus stream habitats within watersheds [28]. Adjacent lake and stream populations in both North America and Europe show substantial genomic divergence, with the repeatability of the loci involved depending on both spatial scale and ecological variation among populations [28,29]. This system thus provides an excellent opportunity to test hypotheses about character divergence at both the level of existing ecotypes and the level of individual loci under selection.

In central Europe, divergence between lake and stream populations has been facilitated by existing genetic variation across multiple lineages. In Switzerland, northeastern Baltic populations with more lake-adapted phenotypes were introduced to the Lake Constance watershed, where they have hybridized with western populations that are better adapted to stream habitats [30–32]. In the <200 years since their initial establishment, multiple islands of genomic differentiation have developed among lake and stream populations, despite overlapping breeding territories [31]. Stream populations have evolved lighter body armour, larger epaxial muscles, lower jaw protrusion and greater prey suctioning capacity [17,18,30]. Whereas lake ecotypes show higher success feeding on zooplankton [17], stream ecotypes more readily strike at benthic chironomids, with capture success decreasing in the presence of macrophytes (E Balmer and RJ Best 2015, unpublished data). Experimental transplants of lake and stream populations into stream habitats in this system have also shown higher survival of fish with stream alleles in stream habitats [33]. If selection on phenotypes and alleles more common in stream ecotypes can be reproduced under specific scenarios of stream prey and habitat complexity, then those conditions should be strong candidate mechanisms to explain the observed rapid evolution of stream ecotypes.

In an outdoor mesocosm experiment, we tested whether habitat complexity (macrophyte presence or absence) and intensity of competition (fish density) affect both the benthic invertebrate prey community and the relative survival and growth of competing lab-reared juvenile sticklebacks. Using a single lake-stream population pair known to be genetically and phenotypically divergent [31], we established mesocosms containing either an equal mix of lake and stream ecotypes or genetically variable lake-stream hybrids (F2s) generated from the same population pair. Including both current ecotype variation and the shuffled genetic variation that makes up this contrast allows us to test whether selective forces consistently explain divergence along the continuum of ecotype formation. We tested the following hypotheses: (1) macrophyte structure should increase benthic invertebrate diversity and abundance by providing increased food and enemy-free space to a greater variety of prey species. (2) Macrophytes should increase average fish growth and condition by sustaining diverse prey, whereas increased competition at higher fish densities should reduce average fish performance. (3a) Macrophytes should disproportionately benefit stream ecotypes by favouring individuals able to obtain benthic prey in a complex benthic foraging arena. (3b) Alternatively, if the foraging arena is unimportant, the increased abundance and diversity of prey in macrophyte-rich environments may weaken differences in performance between ecotypes. (4) Phenotype × environment effects on ecotype performance should be paralleled by genotype × environment effects for divergent loci, so that conditions that favour stream fish over lake fish should also favour F2 hybrids with alleles inherited from a stream parent.

## 2. Material and methods

### (a) Fish rearing

To create lake-stream F2 hybrids, we collected a single F0 pair of stream female and lake male in June 2014, ensuring both were phenotypically typical of their respective populations (see supplementary material, figure S1 for timeline schematic and collection locations). After a year, we randomly bred pairs of F1s to produce a total of 30 separate F2 families, then grouped the families into five groups to supply five experimental blocks. To raise juveniles of each ecotype (lake versus stream) to the same age under the same conditions, we collected and bred 10 pairs each of lake and stream parents from the same lake and stream sites in May 2015.

### (b) Mesocosm experiment

While the fish were growing in the laboratory, in June 2015 we established 50 replicate 1000 l mesocosm environments in 1 m × 1 m × 1.3 m deep plastic tanks (supplementary material, figure S1). The tanks were laid out in a 4 × 13 grid with approximately 1–2 m spacing between tanks, with each sequential set of 10 tanks constituting one spatial block. Within each of the five blocks, 10 treatment combinations were randomly assigned to tanks: the presence (M+) or absence (M−) of macrophytes crossed with fishless controls (F−), low fish density (F+, 30 fish) and high fish density (F++, 60 fish), where fish were either an equal mix of lake and stream ecotypes or a sample of F2s. Each mesocosm began with a layer of sand and gravel, unfiltered water from Lake Lucerne and a mixture of littoral lake sediment from both the lake and stream fish collection sites. We used multiparameter EXO2 sondes (YSI, Yellow Springs, OH, USA) to monitor water quality, confirming that dissolved oxygen was well above stress thresholds for fish (8 mg l^−1^ or higher in all tanks throughout the experiment). See Lürig *et al.* [34] for more details on the establishment of these mesocosms and their subsequent water chemistry and plant communities. We planted macrophytes in half of the tanks in each block (M+), including *Myriophyllum spicatum* plants collected from the Aubach stream and *Chara tomentosa* macroalgae collected from the littoral zone of Lake Lucerne. These field-collected macrophytes also introduced an important diversity of attached benthic fauna. To introduce the same community of benthic invertebrates to the non-macrophyte tanks (M−), we temporarily suspended the equivalent composition of macrophytes in a loose ball at the top of these tanks and ensured all invertebrates had entered the tank before removing it. We also seeded all tanks with zooplankton communities collected from Lake Lucerne. Finally, the juvenile sticklebacks were introduced to the tanks on 2 September 2015.

### (c) Mesocosm sampling

At the end of the experiment, we estimated cover for *Chara*, *Myriophyllum* and filamentous algae separately, with each type capped at 100% cover. Although we did sample zooplankton from all tanks at the start and end of the experiment, we observed that zooplankton (cladocerans and copepods) were reduced to low average densities by the end of the experiment, so we focused on the much more abundant benthos. In each tank, we first used a 30 cm wide 100 µm net to sweep up the middle of each of the four sides of the tank, from sediment to water surface, to sample periphyton and invertebrates. We then used the same net to sweep vertically down from the water surface to collect each *Myriophyllum* plant. Since these plants were taller than the rest of the benthos, this sampling allowed us to capture plant-associated invertebrates without losing them to the filamentous algae. This vertical sampling was conducted in all tanks; only one tank had no *Myriophyllum* at all. Finally, we used the same net to sample one-third of the bottom of the tank in a single 1 m transect through the middle, collecting both benthic producers and consumers. For all of these samples, we separated, dried and weighed the remaining macrophytes according to species and preserved the invertebrates in ethanol. Invertebrates were identified to the lowest possible taxonomic level using taxon-specific determination keys, and body length of each individual was measured under a stereomicroscope with 0.01 mm accuracy to estimate their biomass (see supplementary material, table S1). After identifying and counting all invertebrate taxa, we assigned each species trait values for average biomass (calculated from our data), life cycle duration, locomotion, and body form and flexibility (see supplementary material, table S2). We selected these traits because they are considered to govern the vulnerability of benthic invertebrates to predation by fish [35,36]. Using this trait dataset, we calculated the functional richness of each invertebrate community as convex hull volume (FRic) using the dbFD function from the FD package [37]. We constrained FRic by the total functional richness in the species pool so that it ranges from 0 to 1.

We removed all fish using dip nets, euthanized them with an overdose of MS-222 and froze them individually in water at −80°C. Fish were then thawed for trait measurements and fin clipped for a DNA sample. We assessed performance by measuring final standard length and weight, and regressed weight against length to estimate body condition as the weight each individual would have at a standardized average length. To assess morphological traits relevant to feeding, we took lateral and dorsal photographs of each individual under a dissecting microscope, and analysed these photographs in ImageJ to measure buccal length, gape, jaw protrusion, and epaxial muscle width and height as in McGee *et al.* [16] and Schmid [17].

We identified all surviving ecotype juveniles from the ecotype mesocosms as lake or stream by genotyping each fish and all of the parents used for breeding at nine microsatellite markers [30] and inferring parentage using COLONY software [38]. We recovered 749 survivors out of the 900 original ecotype fish, and all were assigned to a real combination of parents with an average certainty of 94%, and no uncertainty about lake versus stream ecotype. For the F2s, we genotyped juveniles using restriction-site associated (RAD) sequence data (see supplementary material for methods). We recovered 766 surviving F2s, and we obtained sufficient DNA quantity and quality for 642. We filtered for markers where F0s were opposite homozygotes, producing a single expected distribution of F2 genotypes regardless of F1 family. We obtained 557 loci with quality scores over 500.

### (d) Data analysis

We tested for effects of ecosystem treatments on macrophyte cover and invertebrate abundance, biomass and diversity across all mesocosms using general linear mixed models. We set macrophyte treatment (M−/M+), fish treatment (F−/F+/F++) and their interaction as the fixed effects and block as the random effect. In cases with a significant (*p* < 0.05) fish effect, we used Tukey contrasts to test effects of fish presence (F−/F+) versus fish density (F+/F++). All mixed models were implemented using the lmer function of the lme4 package [39] in R [40]. After visual assessment of residuals and transformation of the response variable if needed, we assessed significance of fixed effects using F tests in the Anova function of the car package [41], and random effects using likelihood ratio tests in the lmerTest package [42]. We calculated marginal and conditional *R*^2^ values using the MuMIN package [43] and obtained semi-partial *r*^2^ values as indicators of the relative explanatory power of each fixed effect using the r2beta function of the r2glmm package [44].

We also assessed treatment effects on multivariate invertebrate composition using distance-based redundancy analysis (db-RDA), implemented in the capscale function of the vegan package [45]. We standardized abundances by sample totals and calculated Bray–Curtis community dissimilarities as the response. We set macrophyte treatment (M−/M+), fish treatment (F−/F+/F++) and their interaction as the constraints, and conditioned the permutation assessment by block, evaluating significance of terms in the reduced model by their marginal contribution. To evaluate the effects of ecosystem treatments on invertebrate species traits, we combined RLQ [46] and fourth-corner analysis [47]. The multivariate RLQ method, implemented in the ade4 package [48], was used to summarize and visualize associations between mesocosm treatments and the traits of the invertebrate community. The fourth-corner analysis, performed using the traitglm function from the mvabund package [49], was used to test the statistical relationships between species traits and treatments. We constrained the significance test in the fourth-corner model by performing 1000 bootstrap resamples within blocks.

We tested fish survival rate using a glmm implemented in the glmer function with a binomial link, and after confirming equidispersion, assessed significance of fixed effects using Wald chi-squared tests. We tested final length as a proxy of growth (given that we minimized initial length variation within mesocosms), and final condition using lmms in lmer. For all variables, we first ran models including all mesocosms with macrophyte treatment (M−/M+), fish treatment (F−/F+/F++), fish type (ecotypes/F2s) and their interactions as the fixed effects and block as the random effect. We then conducted these tests separately for the ecotypes and the F2 hybrids. When there were multiple values per mesocosm (i.e. many fish or two ecotypes), we included mesocosm ID as a second random effect.

To test for differences in the performance of fish with stream versus lake alleles, we used QTL mapping to identify loci related to final length and weight in the F2 tanks. We did not identify loci associated with survival because the fish were too small to sample tissue for genotyping at the outset of the experiment. In addition to final length and weight, we tested for feeding-related mechanisms for differential performance using QTL analysis for size-corrected oral morphology traits. After building a linkage map (see supplementary material), we used the qtl package v1.52 [50] in R with extended Haley–Knott regression to analyse 613 F2 hybrid individuals and 454 markers. We evaluated QTL effects for each trait, first testing only for phenotype-to-genotype associations, and then including macrophytes (M−/M+) or fish density (F+/F++) as an additive or interactive covariate. We ran 1000 permutations for each model, used an alpha value of 0.05 to identify significant markers and quantified the percentage of trait variance explained using the fitqtl function.

## 3. Results

### (a) Ecosystem effects on prey availability

We successfully established a contrast in benthic habitat structure, with final macrophyte cover (composed of *Chara* spp. and *Myriophyllum* spp.) averaging 32% of the total area in M+ tanks and 3% in M− tanks. We identified 8917 invertebrate individuals belonging to 45 taxa (see supplementary material, table S1). As predicted, we found that macrophyte presence increased macroinvertebrate biomass, species richness and functional richness (figure 1, table 1). The positive effect of macrophytes was consistent, regardless of fish presence or density (table 1, no significant M×F interactions). Fish predation did independently decrease macroinvertebrate abundance but not total prey biomass or richness (figure 1). In fact, high fish density mesocosms had higher invertebrate species richness than mesocosms with low fish density (figure 1c). This may be partly owing to gape limitation in the slower-growing high-density fish.

**Figure 1.**
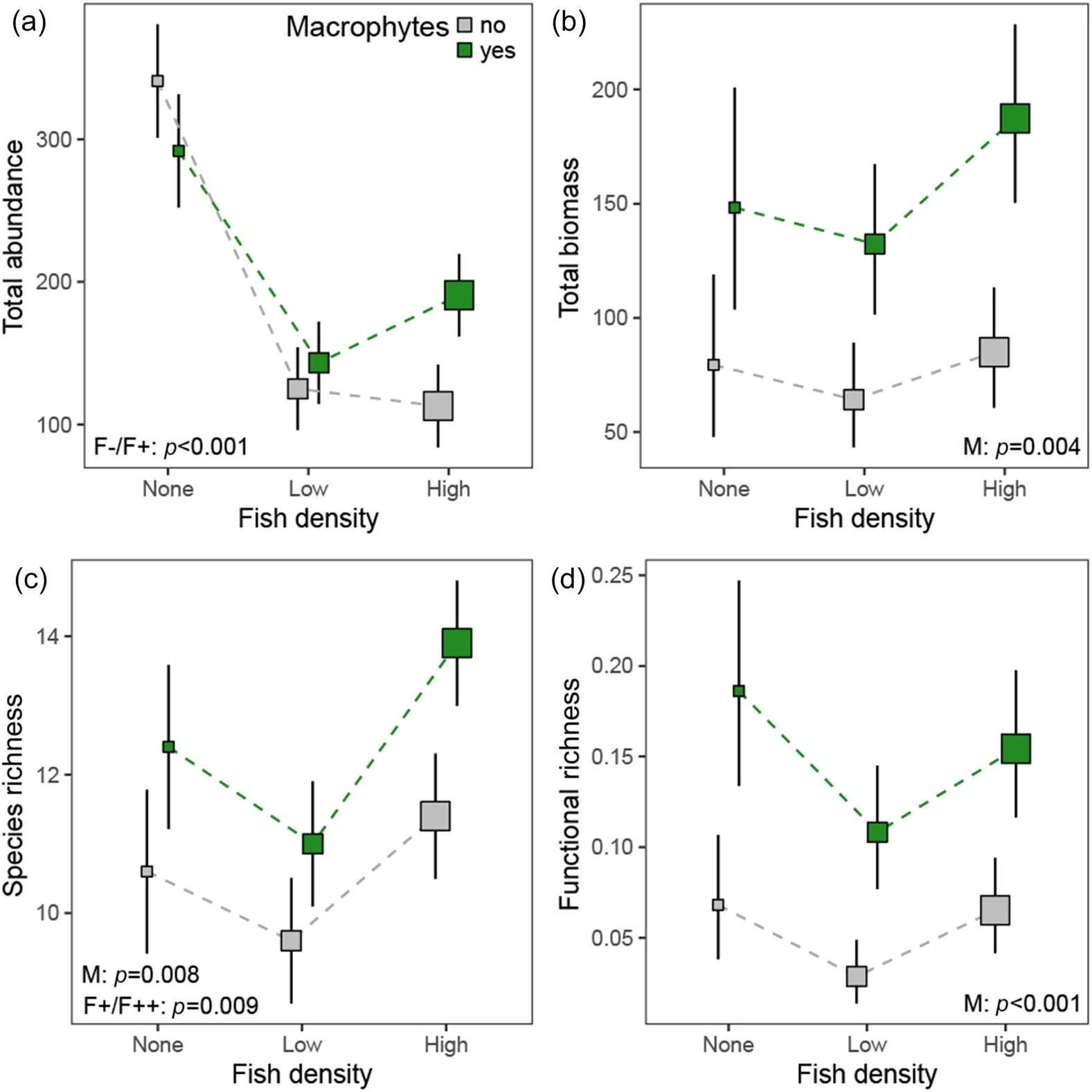
Effects of fish density and macrophyte structure on benthic invertebrate communities, including (a) total abundance, (b) total biomass, (c) species richness and (d) functional richness. Symbol colour indicates macrophyte presence and symbol size indicates fish treatment. Significant effects of macrophytes (M), fish presence (F−/F+) or fish density (F+/F++) are annotated in each panel with *p*-values from table 1, and error bars are +/−1 s.e.

**Table 1.** Ecosystem treatment effects on macroinvertebrates assessed using linear mixed-effects models (abundance, biomass, species richness, functional richness), db-RDA (community composition) and fourth-corner analysis (species traits). Effects with *p* ≤ 0.05 are bolded to indicate significance. Models with significant fish effects were evaluated using pairwise contrasts to identify whether those effects were driven by fish presence or fish density. Test statistics (stat) represent *f* ratios (general linear mixed models), pseudo-F ratios (db-RDA) and differences in deviance between full and reduced models (fourth-corner analysis). Degrees of freedom (d.f.) refer to the numerator and denominator d.f. for *f* ratios (mixed models and db-RDA) and the difference in degrees of freedom between nested models (fourth-corner analysis). Semi-partial *r*^2^ values indicate the relative explanatory power of each fixed effect. *R*^2^ denotes the marginal coefficient of determination in mixed models and the variation explained by treatments in db-RDA. A block effect was included in all models either as a random effect (mixed models), a covariate (db-RDA) or as a restriction during resampling tests (fourth-corner analysis).

response	fixed effect	stat	d.f.	*p*	partial *r*^2^	*R*^2^
abundance	macrophytes (M−/M+)	1.38	1,40	0.247	0.015	0.42
	**fish (F**−**/F+/F+**+)	16.46	2,40	**<0.001**	0.338	
	M × F	1.87	2,40	0.168	0.067	
	**fish presence (F**−**/F+)**			**<0.001**		
	fish density (F+/F++)			0.801		
biomass^*^	**macrophytes (M**−**/M+)**	9.50	1,40	**0.004**	0.027	0.18
	fish (F−/F+/F++)	0.74	2,40	0.483	0.008	
	M × F	0.09	2,40	0.916	0.004	
species richness	**macrophytes (M−/M+)**	7.88	1,40	**0.008**	0.023	0.23
	**fish (F**−**/F+/F**+**+)**	4.72	2,40	**0.014**	0.045	
	M × F	0.26	2,40	0.770	0.009	
	Fish presence (F−/F+)			0.400		
	**fish density (F**+**/F++)**			**0.009**		
functional richness^*^	**macrophytes (M−/M+)**	17.79	1,40	**<0.001**	0.064	0.26
	fish (F−/F+/F++)	2.57	2,40	0.089	0.042	
	M × F	0.07	2,40	0.936	0.002	
community composition	**macrophytes (M**−/**M+)**	7.86	1,40	**0.001**	0.140	0.32
	**fish (F**−**/F+/F**++)	3.686	2,40	**0.001**	0.131	
	M × F	1.59	2,40	0.111	0.057	
	**fish presence (F**−**/F+)**			**0.001**		
	fish density (F+/F++)			0.303		
species traits	**macrophytes (M**−/**M+)**	42.36	10	**0.023**		
	**fish (F**−**/F+F++)**	115.7	20	**0.002**		
	M × F	22.34	20	0.639		
	**fish presence (F**−**/F+)**			**0.002**		
	**fish density (F**+**/F++)**			**0.002**		

* These variables were square-root transformed to meet assumptions of normality in model residuals.

Fish and macrophyte presence also independently shaped macroinvertebrate community composition, including species’ relative abundances (figure 2a) and traits (figure 2b,c, table 1). As fish density increased, invertebrate communities shifted towards species with cylindrical shapes, higher body flexibility and burrowing behaviour (figure 2b), traits which probably confer tolerance of predation by high densities of small fish. Regardless of fish density, structurally complex habitats with macrophytes were associated with species with higher biomass, streamlined body shape and walking locomotion (figure 2c). These characteristics may have been facilitated by refuge from invertebrate and/or fish predators or by higher food quality for these species in the community of epiphyton growing within vegetation.

**Figure 2.**
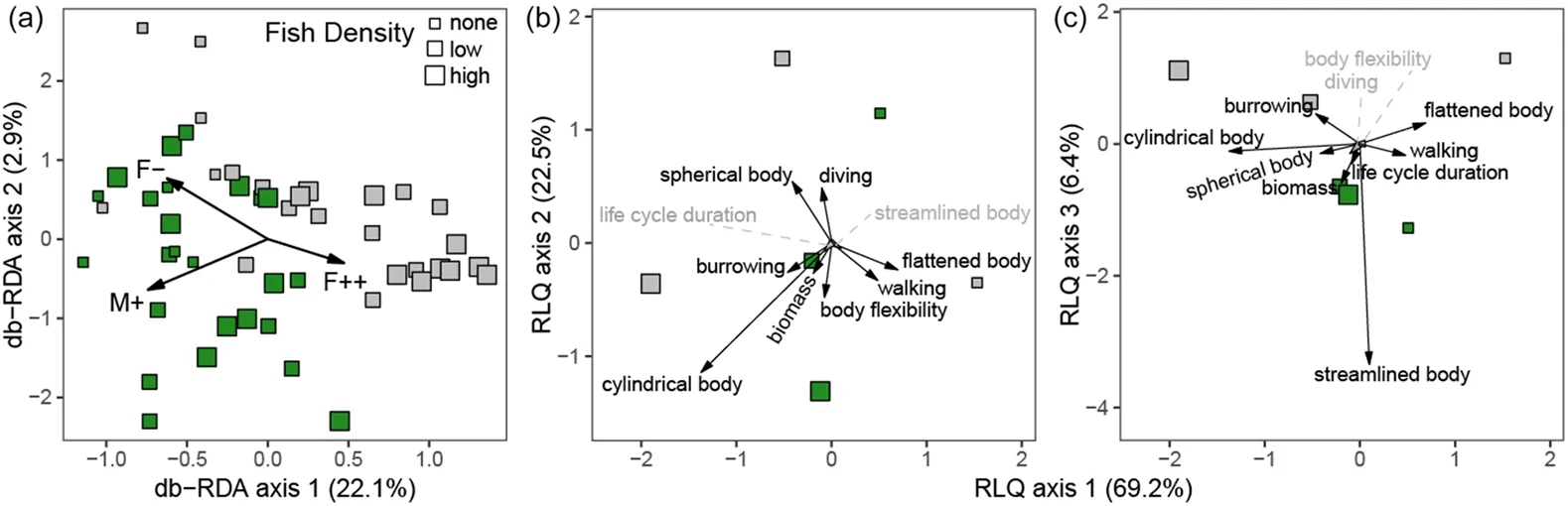
Effects of fish density and macrophyte presence on (a) species composition, and (b, c) species traits of benthic invertebrate communities. Symbol colour indicates macrophyte treatment and size indicates fish density. In the db-RDA plot (a), sample scores (squares) are shown along with macrophyte (M+) and fish (F−/F++) contrasts. In the RLQ plots (b,c), treatment centroids (squares) are displayed alongside species trait vectors. For brevity, only traits with canonical weights greater than 0.2 are shown, and the smallest two vectors in each panel are greyed out. The variability explained by the ordination axes is given in parentheses. For statistics, see table 1.

### (b) Ecosystem effects on fish survival and performance

As predicted, higher fish densities strongly decreased survival, final length and condition of both ecotypes and F2 hybrids, almost certainly owing to higher competition for accessible food (figure 3). Because fish type (ecotypes versus F2s) had significant interactive effects on survival and main effects on condition, we present the separate results for these two groups. Macrophyte presence countered the negative effect of density to consistently increase average fish condition, at least at high density (figure 3c,f), but increased survival only in F2s (figure 3d). Thus, the benefits of higher prey availability and diversity in vegetated habitats are mediated by both the density and the phenotypic composition (ecotypes versus F2 hybrids) of competing predators.

**Figure 3.**
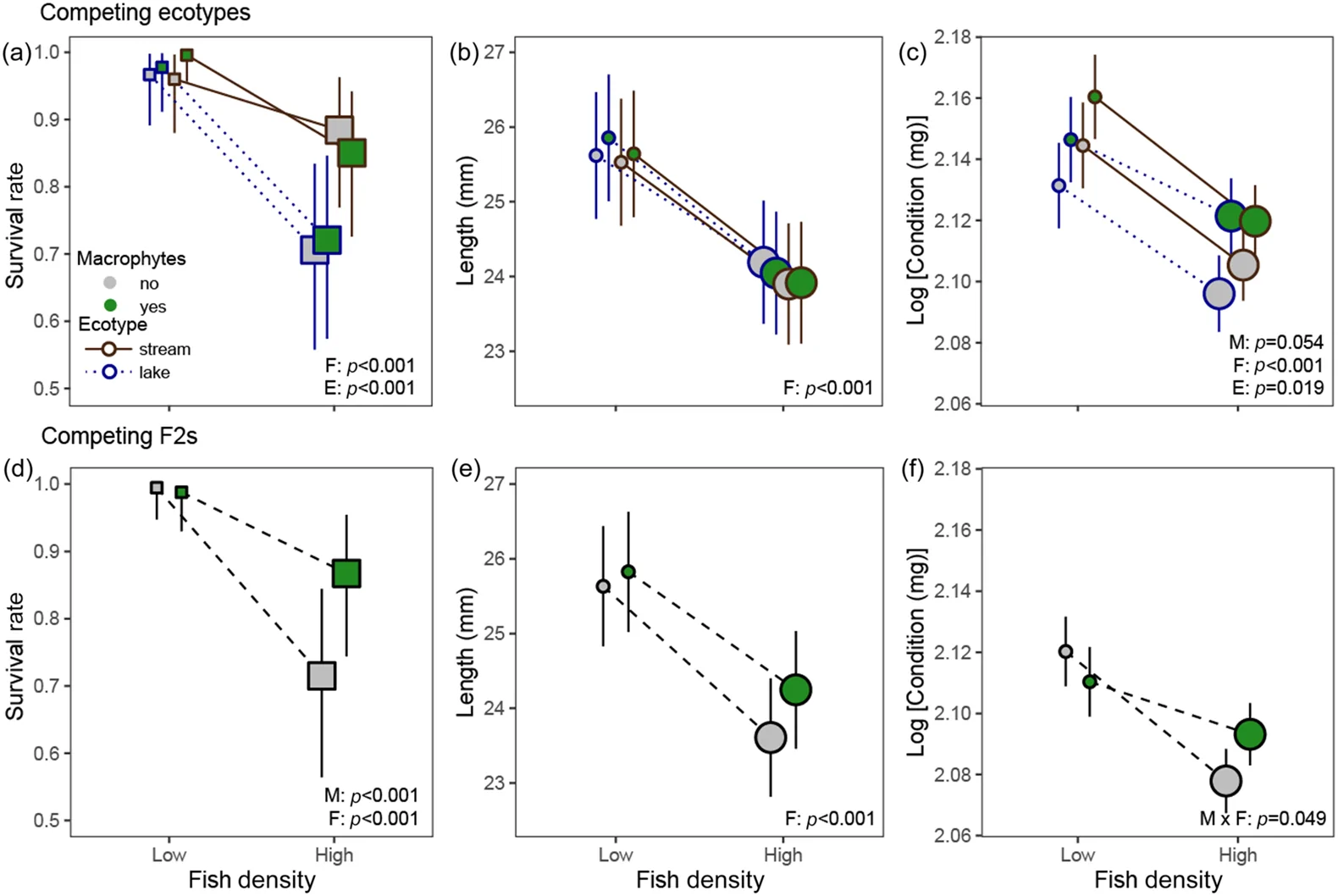
Effects of fish density and macrophytes on (a,d) total fish survival, (b,e) final length as an indicator of growth and (c,f) condition (log_10_-transformed weight per standardized length). Significant effects of macrophytes (M), fish density (F), ecotype (E, lake versus stream in panels a–c only) or their interactions are annotated in each panel. Error bars are 95% confidence intervals. Square symbols are averages of mesocosm-level data; circles are averages of fish-level data. All models for fish-level data (length and condition) included a significant random effect of mesocosm (*p* ≤ 0.002). For full statistical results, see supplementary material, table S3.

### (c) Ecotype performance

Stream fish had higher survival and condition than lake fish (figure 3a,c), despite no higher growth (figure 3b). However, contrary to our hypothesis, these advantages were not affected by macrophytes (no interactive effects between macrophytes and ecotype, figure 3a,c). The survival advantage for stream fish was most obvious at high density, when mortality rates were substantial, and declined to neutral at low density where survival was near 100% (figure 3a, supplementary material, figure S2). Thus, the survival advantage of the benthic stream fish was maximized by resource scarcity rather than by a higher richness of the benthic prey community or an ability to navigate and forage in complex habitats.

### (d) Genotype performance

The F0 parents crossed for this experiment were genetically distinct and representative of the stream versus lake ecotypes in this system (supplementary material, figures S3, S5). The linkage map included 454 individual loci across 21 chromosomes (supplementary material, table S4, figure S4). QTL analysis identified a significant performance advantage for three alleles coming from the stream parent, each contributing a 3%–4% increase in final length. The QTL explaining the highest variance in final length and weight was on chromosome 16, where fish homozygous for the allele inherited from the stream parent had higher average final length by approximately 1 mm (figure 4, supplementary material, table S5). This allele is not fixed in the stream population, but was twice as frequent among the stream fish collected to be parents of the ecotypes compared with the lake parents, and was only ever homozygous in stream parents. We also identified two loci on chromosomes 10 and 12 where fish homozygous for the stream allele were again an average of 1 and 0.75 mm longer, respectively, but only at low density where the scope for growth was higher (see supplementary material, table S5). Just as macrophyte presence did not alter the condition advantage of the stream ecotype, macrophyte treatment did not modify the genetic effects of the QTLs for final length. We did not identify any significant QTLs for condition.

**Figure 4.**
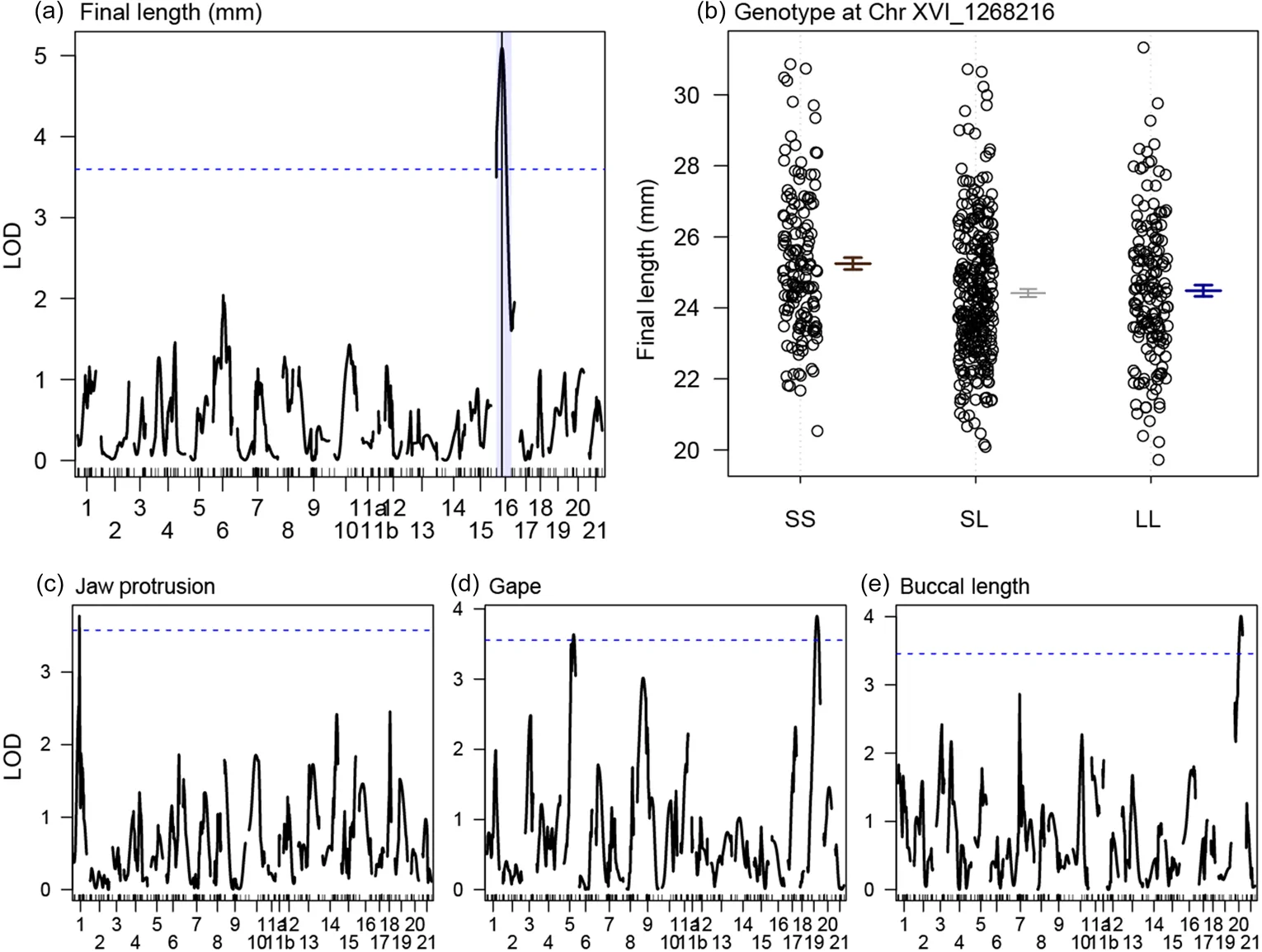
Joint effects of QTL markers and fish density on growth (final length at the end of the experiment) and mouth morphology of F2 fish. Dashed blue lines show the genome-wide significance threshold for LOD (logarithm of odds) values for 1000 permutations against an alpha of 0.05. (a) The best predictor of final length and weight regardless of fish density was a single marker on chromosome 16. (b) Fish that were homozygous for the stream parent's allele at this locus had a higher final length than either of the other two genotypes. Significant QTLs for size-corrected (c) jaw protrusion, (d) gape and (e) buccal length were also identified; these were not dependent on fish density.

In addition to QTLs for length and weight, we found a few loci significantly affecting size-corrected feeding morphology, including jaw protrusion, gape and buccal length (figure 4). These traits covered similar distributions in the F2 juveniles as they did in the ecotype juveniles (see supplementary material figure S5). The allele inherited from the lake parent increased jaw protrusion and the allele inherited from the stream parent increased buccal length (see supplementary material, table S5). None of these loci associated with feeding morphology were directly predictive of final length. For gape, the phenotypic effect of having the stream versus lake allele differed between the two QTLs identified (see supplementary material, table S5).

## 4. Discussion

We hypothesized that replicating the vegetated structure of stream and littoral habitats where benthic sticklebacks are found would support a richer prey community of benthic invertebrates, which could either strengthen or dampen selection for stream phenotypes. We found that macrophyte habitats had strong positive effects on benthic prey communities, including biomass, richness (figure 1) and the incidence of particular macroinvertebrate traits (figure 2). Interestingly, these prey effects did not translate into consistent effects on stickleback performance. Macrophytes increased total survival and average body condition in some circumstances (figure 3), but there were no significant interactions between macrophyte presence and ecotype or genotype. The competitive advantage of stream phenotypes came not from their greater ability to find and capture diverse benthic prey in a complex vegetated habitat, but from their ability to find and use any food under strong resource limitation. In fact, the higher prey availability supported by complex habitats may have dampened selection by increasing average body condition across all phenotypes (figure 3c,f). In contrast, low resource availability may benefit benthic phenotypes better adapted to access either specific scarce resources or a wider range of benthic resources than lake ecotypes.

### (a) Habitat structure supports a diverse prey community

Relative to open water or bare substrate, vegetated habitats can accommodate a more complex community of invertebrate species with a variety of hiding and escape strategies [6,19]. This is consistent with our finding of increased species and functional richness in vegetated mesocosms (figure 1). Even in mesocosms without fish, the macrophyte habitat may have increased invertebrate richness by increasing food resources [20] and sheltering prey from predaceous insects such as beetles and damselflies. In particular, macrophytes can facilitate species whose size, behaviour or pigmentation would otherwise make them vulnerable to predation [51]. For example, finely branched macrophytes such as *Myriophyllum* can increase densities of large species such as annelid worms and odonates [52]. We found a similar pattern, with macrophyte presence supporting invertebrate taxa with higher biomass and streamlined body shapes (figure 2b,c).

### (b) Competition intensity constrains access to prey

The higher invertebrate richness we found in macrophyte mesocosms could correspond to higher quality food resources benefiting individual fish performance [22,53] and resulting in increased condition of the high-density competitors (figure 3c,f). However, the shift towards larger-bodied taxa in both vegetated habitats and under higher fish density (figure 2b,c) may actually have constrained growth in those environments. Higher density strongly decreased fish growth (figure 3b,e), which in turn limited individual gape size, making larger invertebrate taxa and individuals inaccessible as prey. The higher prey richness in vegetated habitats should allow for greater feeding niche width and specialization [7], but only if that diversity of prey is accessible to consumers. Here, larger prey may have only been accessible to fish in the low-density mesocosms with less competition, where it is possible that early access to sufficient zooplankton and small benthic prey facilitated an early growth spurt regardless of habitat type. Feeding early in ontogeny can have long-term effects on adult size and performance [54], and the first feeding activity after migration into a new habitat can also shape the prey size spectrum throughout the season [55]. Combined with this previous work, our results suggest that growth rates and ontogenetic shifts can interact with the overall rate of resource depletion to determine whether competition promotes or constrains divergence in feeding niche [11].

### (c) Benthic fish survive when resources are limited

Juvenile sticklebacks can reach high densities in vegetated habitats when small fish seek refuge from predation [56] or pursue higher prey abundance [24]. Here, we found that stream fish clearly had a higher survival and condition than the lake fish they competed with, regardless of benthic habitat complexity (figure 3a,c). Unlike early work showing clear growth advantages for benthic over limnetic sticklebacks in benthic habitats [14], stream fish did not grow to larger final lengths or larger gapes, although they did differ in some aspects of feeding morphology (see supplementary material, figure S5). This may be because they invested more resources in musculature or fat, attaining higher condition in all environments (figure 3c). As a result of equivalently low growth and gape, high-density stream fish should have had access to the same limited proportion of the prey size spectrum as the lake fish. Their higher survival despite this scarcity is consistent with several previous studies. In a selection experiment in the wild using multiple Lake Constance stream populations, stream juveniles showed maximum advantage over lake juveniles at high densities [33]. Sticklebacks from a more benthic lineage (upper Rhone) also outperformed those from the northeastern limnetic lineage in mesocosms with depleted prey, regardless of whether the competing fish were young juveniles [57] or adults [58]. Although none of these experiments used or reproduced the fully pelagic habitats that we would expect to maximize the performance of lake ecotypes, the effects on relative stream advantage are consistently related to fish density rather than the specific complexity or quality of the benthic prey or habitat.

Instead of being tightly adapted for foraging for particular food, stream fish may be excellent generalists because they encounter a diverse prey base in their natural habitat. Benthic prey are extremely variable in size, shape and habitat use, including everything from very small mobile prey like ostracods to large burrowing worms to swimming and crawling insect larvae [59], and sticklebacks in benthic environments transition between these very different prey sizes as they grow [60]. In contrast, lake sticklebacks have evolved to be better at capturing the much more uniformly small and mobile zooplankton in an open water habitat [17], which can make up the majority of their diet in the wild [61]. We did not detect any direct correlations between prey functional richness and the relative performance of stream fish, but characterizing the diversity of the prey items accessible to fish may require more extensive modelling of treatment-specific gape limitation and how that changes with growth.

Alternatively, stream fish could be better than lake fish at capturing a particular type of prey that is most available during lean times. This type of specialization can evolve when seasonal bottlenecks in resource availability are particularly important for determining survival [4]. Fish populations in small streams with large seasonal fluctuations in water temperature, macrophyte growth and the availability of appropriately sized insect larvae [59] might well be limited by the scarcest season. An ability to access alternative prey under strong competition will not lead to higher survival if those prey are of insufficient abundance or quality to equal the cost of their capture and handling [62]. However, stream fish may also be metabolically or physiologically more efficient, accomplishing equivalent growth to lake fish even with fewer resources or higher parasite loads [63]. Evidence from other systems has shown that lake-stream divergence in threespine stickleback is less parallel than the divergence observed between sympatric limnetic and benthic forms [64]. This contrast highlights the multiple dimensions of selection that may differ between any single lake and stream habitat within a watershed, compared with lake populations that can move between limnetic (open water) and littoral (shoreline) habitats throughout the year to optimize abiotic and biotic conditions [61].

### (d) Identifying genetic and phenotypic drivers of divergence

Experiments that can detect genetic determinants of fitness correlates like growth or condition are valuable because they support our inference of evolutionary mechanisms for ecological character divergence [26,65]. Identifying the genetic basis of phenotypes that can shape and be shaped by ecological variation also informs our understanding of the potential rate and directions of eco-evolutionary dynamics [66]. Here, parallel to the higher survival and condition of stream ecotypes, we found that F2 juveniles homozygous for alleles inherited from the stream parent at three QTL loci achieved larger final length and weight, at least at lower densities where substantial growth was possible. For the locus with the strongest effect, the allele was also twice as frequent among our sample of stream ecotype parents than among the lake fish, suggesting it could be associated with ecotype divergence in the wild. However, we did not find the genotype-by-environment interactions we expected to affect the performance of phenotypes that have evolved in contrasting environments. The advantage of the stream fish and genotypes did vary with the density of competitors, but was not affected by habitat treatment. In addition, the chromosomes where we found loci associated with growth (16, 10 and 12) have previously shown QTLs associated with traits such as body shape, feeding and defence, whereas loci predictive of adult body size have been located on the sex chromosome [67]. Given that genetic determinants of growth rates can change through ontogeny [68], such differences between studies on adults and our findings for juveniles should not be surprising.

Depending on the environment, juvenile growth could involve loci that are functionally connected to a wide range of traits, from physiological determinants of metabolism, to growth hormones, to morphological traits related to food capture. Condition (weight per length) may depend on an even greater number of loci related to both growth rate and the distribution and size of muscle and fat, which could explain why we did not find any significant QTLs for this measure of performance. For this reason, moving beyond individual loci to track changing patterns of overall heterozygosity through selection can also be informative. Across multiple F2 crosses between contrasting ecotypes of threespine stickleback, ecological pressures in experimental pond environments have consistently selected for heterozygotes over fish with opposite ancestry homozygous genotypes across loci [69]. This finding suggests that multi-locus incompatibilities can cause disadvantages in an ecological context. However, we did not find elevated heterozygosity in the high-density tanks, where mortality was high compared with the low-density treatment.

Interactions between allelic effects and fish density highlight the complexity of adaptation to habitats that differ in multiple abiotic and biotic dimensions. For example, in a previous selection experiment in threespine stickleback, the frequencies of armour phenotypes and loci linked to those phenotypes diverged under the presence versus absence of a top predator [65]. Importantly, selection was mediated not directly by the success of predation attempts, but by predator effects on stickleback population density (competition intensity) and habitat use (hiding in benthic vegetation) [65]. This interplay between top-down effects on fish density and bottom-up availability of resources driving character divergence has been demonstrated in both stickleback [25] and Trinidadian guppies [70]. Despite the complexity, these findings emphasize the importance of explicitly including density dependence in selection experiments [71].

Adaptation to different combinations of competition, predation and habitat probably involves many traits and even more genetic loci. Here, we found a few loci associated with juvenile feeding morphology such as gape size and jaw protrusion, but none of these were associated with variation in growth. The patterns we found at these loci, where homozygous stream alleles were associated with less jaw protrusion but greater buccal length, are broadly consistent with the significant phenotypic differences between benthic and limnetic ecotypes in central European lineages [30]. In this and other stickleback systems, benthic and limnetic populations have diverged in feeding, defence and swimming traits [18,31,72], which could all be relevant to finding and capturing food resources in environments with different physical structure and predation risks. However, the ultimate value of all of these traits for survival is probably strongly dependent on growth [73], and likely to change through ontogeny with the match between resource availability and individual gape.

## 5. Conclusions

In this experiment, macrophytes clearly facilitated a diverse prey community and high total prey biomass, but this did not promote ecological divergence in juvenile threespine stickleback at either the ecotype or genotype level. Instead, by increasing average condition and survival, macrophyte complexity probably weakened differential selection. Furthermore, even though macrophytes had the largest effect on many aspects of the invertebrate community, fish density had the strongest effect on fish survival and condition. This finding reinforces the importance of density dependence as a primary driver of performance differences between habitats [70].

Even though we did not detect the effects of aquatic macrophytes on the performance differential among stickleback phenotypes or genotypes, these plants had strong ecological effects on the prey community. Especially over longer timescales including seasonal pulses of resource availability, aquatic vegetation can contribute substantially to the maintenance of fish populations and fish species diversity [53], in addition to stabilizing ecosystem states [74]. Restoring and maintaining aquatic vegetation, which is threatened by both eutrophication and climate change, is clearly important to support the community-level diversity that generates ecosystem services in aquatic ecosystems [75]. The importance of habitat complexity for maintaining adaptive capacity or population divergence within aquatic species remains unclear, but should be investigated at a range of both prey community types and predator densities.

## Supplementary Material



## Data Availability

Raw sequence data are available at SRA BioProject PRJNA789317 [76]. All other data and analysis scripts are available at [77]. Supplementary material is available online [78].
