# Supplementary material for: Aquatic vegetation promotes prey diversity but not the diversification of predator phenotypes
Source: Proc Biol Sci. 2026 Oct 14;293(2080):20261458. doi: 10.1098/rspb.2026.1458 (PMC13678722; doi:10.1098/rspb.2026.1458)
Supplement: Supplementary file 1 [file rspb.2026.1458.zip › rspb.2026.1458/rspb20261458_si_001.docx]

**Supplementary Material for** Best, R.J., M. Svitok, M.D. Lürig, C.M. Hudson, V. Dekan Carreira, M. Novikmec, M.C. Leal, O. Seehausen, P.G.D. Feulner, and B. Matthews. Aquatic vegetation promotes prey diversity but not the diversification of predator phenotypes. Proceedings of the Royal Society: B. DOI: 10.1098/rspb.2026.1458.

**A. Supplementary methods**

**Breeding**

To study divergence of lake and stream ecotypes within this system, we focused on two parapatric populations in eastern Lake Constance that have some overlap in their breeding territory (lake adults return to the stream outlet to reproduce), but also show significant genomic divergence broadly aligned with several known QTLs in threespine sticklebacks (Marques et al. 2016). Collection sites were Aubach stream at Oberriet (47.327874 N, 9.578103 E) and Lake Constance at Altenrhein (47.498611 N, 9.556944 E).

**Figure S1:** a) Timeline for breeding of juvenile F2s and ecotypes from wild-collected lake and stream individuals, and establishment of replicate mesocosm ecosystems. Parents were bred in the lab using artificial (in vitro) fertilization. Both the F2s and the ecotype juveniles hatched between 19 May and 12 June 2015, were spread across multiple aquaria to achieve similar densities, and were fed artemia nauplii and zooplankton for 2.5–3 months before their introduction to the mesocosms. b) Factorial experimental design crossing macrophyte presence (M-/M+) with fish presence (F-/F+) and density (F+ = 30 fish vs. F++ = 60 fish per mesocosm). Fish mesocosms were stocked with either F2s or an equal mix of lake and stream ecotypes, all approximately three months old. At the time of stocking (September 2, 2015), we assembled juveniles in 12 groups of 30 fish for each block, maximizing initial size similarity across and within groups. We photographed each group from above while fish were swimming in straight lines, and randomly assigned one group to each low fish density tank and two groups to each high fish density tank. We used lengths extracted from the images to confirm no initial differences in average fish length among treatments or fish type (F2s versus stream versus lake).

**F2 genotyping**

Juveniles were allocated to libraries based on their experimental block, so that each library included 95 fish sampled across all mesocosm treatments. In addition, we obtained high coverage sequencing for the F0 parents by including them in four of the libraries and combining the reads. Finally, to compare the genomic identity of the F0s to the original lake and stream source populations and the lake and stream ecotype juveniles present in the ecotype mesocosms, we also included all 40 wild-caught lake and stream parents of those juveniles (20 parents of each ecotype).

Following DNA extraction using DNeasy Blood and Tissue kits (Qiagen, Germany), DNA concentrations were assayed using Qubit® dsDNA High Sensitivity Assay Kits (Life Technologies) and normalized to a concentration of at least 10 ng/μl. RADseq libraries were then prepared by Floragenex (Eugene, Oregon) using a single digest protocol with the Sbfl restriction enzyme. This included seven libraries sequenced on an Illumina HiSeq in 2016, and two additional libraries sequenced on an Illumina NextSeq500 in 2017. The first seven libraries produced 163-175 million 100 bp reads each. After processing these we re-sequenced 30 of the 642 mesocosm F2s (originating from several different mesocosms) as part of two NextSeq500 runs to increase sequencing depth.

For each of the libraries, after receiving fastq files from Floragenex we ran library quality checks with FastQC v0.11.4 [(Andrews 2015)](https://paperpile.com/c/YSQqEs/nUwY), filtered reads to include only those with an intact Sbf1 restriction site (89-93% of reads) and de-multiplexed and barcode-trimmed reads using process_radtags module in Stacks v1.37 [(Catchen et al. 2013)](https://paperpile.com/c/YSQqEs/ITTu). We aligned reads to the Glazer et al. [(Glazer et al. 2015)](https://paperpile.com/c/YSQqEs/YtzB) reference genome using end-to-end alignment in Bowtie 2 v2.2.6 [(Langmead and Salzberg 2012)](https://paperpile.com/c/YSQqEs/Fc4u) with default parameters, mapping 92-93% of the retained reads in each library. In each library we obtained >20,000 loci with a mapped depth of >20.

To call genotypes, we first identified sites where the F0s were opposite homozygotes, producing all heterozygote F1 genotypes and therefore a single expected distribution of F2 genotypes regardless of F1 family. We used HaplotypeCaller in GATK 3.5 [(Poplin et al. 2018)](https://paperpile.com/c/BWToip/GxdzC) to call genotypes for the parents, then used vcftools 0.1.15 [(Danecek et al. 2011)](https://paperpile.com/c/BWToip/iahIb) to retain only those sites with exactly two non-reference alleles, no heterozygotes, no missing data, and a depth of >20 for each F0. We then called genotypes for all other individuals at these sites, and used vcftools to retain sites with no more than 20% missing data, no indels, and average depth of >20.

**Linkage mapping for QTL analysis**

To build a linkage map for QTL mapping, we defined lake and stream alleles as those present in the respective F0, and coded all F2s as lake homozygote, heterozygote, or stream homozygote using R to translate formats from vcf. We input genotypes into JoinMap 4 (Stam 1993), excluding 18 loci mapped to an unresolved chromosome in the reference genome, 22 loci identical to each other, and 69 loci with extreme segregation distortion, retaining 454 loci. Using an LOD threshold of 14, these loci grouped into scaffolds consistent with their mapped chromosome with the exception of chromosome 11, which accounted for two groups. We mapped the resulting 22 chromosomes using the regression mapping procedure with Haldane’s function. We excluded 29/642 fish (two individuals with more than 30% missing data and 27 with high crossover frequencies, indicating genotyping errors.

**References**

Andrews, S. 2015. FastQC: A Quality Control Tool for High Throughput Sequence Data. <http://www.bioinformatics.babraham.ac.uk/projects/fastqc/>

Catchen, J., P. A. Hohenlohe, S. Bassham, A. Amores, and W. A. Cresko. 2013. Stacks: an analysis tool set for population genomics. Molecular Ecology 22:3124–3140.

Danecek, P., A. Auton, G. Abecasis, C. A. Albers, E. Banks, M. A. DePristo, R. E. Handsaker, G. Lunter, G. T. Marth, S. T. Sherry, G. McVean, R. Durbin, and 1000 Genomes Project Analysis Group. 2011. The variant call format and VCFtools. Bioinformatics 27:2156–2158.

Glazer, A. M., E. E. Killingbeck, T. Mitros, D. S. Rokhsar, and C. T. Miller. 2015. Genome Assembly Improvement and Mapping Convergently Evolved Skeletal Traits in Sticklebacks with Genotyping-by-Sequencing. G3 Genes|Genomes|Genetics 5:1463–1472.

Langmead, B., and S. L. Salzberg. 2012. Fast gapped-read alignment with Bowtie 2. Nature methods 9:357–359.

Poplin, R., V. Ruano-Rubio, M. DePristo, T. Fennell, M. Carneiro, G. Van der Auwera, D. Kling, L. Gauthier, A. Levy-Moonshine, D. Roazen, K. Shakir, J. Thibault, S. Chandran, C. Whelan, M. Lek, S. Gabriel, M. Daly, B. Neale, D. MacArthur, and E. Banks. 2017. Scaling accurate genetic variant discovery to tens of thousands of samples. <https://doi.org/10.1101/201178>

Stam, P. 1993. Construction of integrated genetic linkage maps by means of a new computer package: Join Map. The Plant Journal 3:739–744.

**B. Invertebrate identification, biomass, and traits**

*Please see all references for identification, length-weight equations, and trait determinations following these tables.*

**Table S1:** Length-mass equations for all invertebrate taxa.

| **Taxon** | **Developed for** | **equation: W (mg DM) =** | **source** |
| --- | --- | --- | --- |
| Ablabesmyia cf. phatta | Ablabesmyia sp. | 0.0010*L^2.884 | Benke et al. 1999 |
| Asellus aquaticus | Asellus aquaticus | 0.002029431*L^1.14 | Baumgärtner & Rothhaupt 2003 |
| Athripsodes atterimus | Athripsodes atterimus | 0.014122302*L^1.07 | Baumgärtner & Rothhaupt 2003 |
| Caenis horaria | Caenis sp. | 0.005092431*L^0.21 | Baumgärtner & Rothhaupt 2003 |
| Ceratopogonidae | Ceratopogonidae | 0.016082879*L^1.12 | Baumgärtner & Rothhaupt 2003 |
| Cladopelma lateralis gr. | Cladopelma spp. | 0.0010*L^2.606 | Benke et al. 1999 |
| Cladotanytarsus mancus gr. | Taytarsus spp. | 0.0012*L^2.294 | Benke et al. 1999 |
| Clinotanypus sp. | Taypodinae | 0.0026*L^2.503 | Benke et al. 1999 |
| Cloeon dipterum | Cloeon dipterum | 0.0004*L^3.793 | Svitok, unpubl. |
| Coenagrion puella | C. pulchellum | 0.0918*L^2.10 | Pavlov & Zubina (1990) |
| Corynoneura scutellata gr. | Corynoneura sp.(lobata) | (0.00147*L^2.1)/(1-0.042) | Nolte (1990) + Benke et al. 1999 (AFDM conversion) |
| Cryptochironomus spp. | Chironomus spp. | 0.00068*L^2.620 | Benke et al. 1999 |
| Cypridopsis vidua | Cypridopsis | 0.027112639*L^2.113 | Anderson et al. 1998 |
| Demicryptochironomus spp. | Chironomus spp. | 0.00068*L^2.620 | Benke et al. 1999 |
| Dicrotendipes spp. | Dicrotendipes sp. | 0.00059*L^3.14 | Benke et al. 1999 |
| Endochironomus cf. tendes | Chironomus spp. | 0.00068*L^2.620 | Benke et al. 1999 |
| Ephemera danica | Ephemera danica | 0.00316*L^2.86 | Tokeshi 1985 |
| Erpobdella octoculata | Erpobdella octoculata | 0.040762204*L^2.22 | Edwards et al. 2009 |
| Erpobdella spp. juv. | Erpobdella octoculata | 0.040762204*L^2.22 | Edwards et al. 2009 |
| Glossiphonia nebulosa | G. complanata | 0.120031629*L^2 | Edwards et al. 2009 |
| Glossiphonia spp. juv. | G. complanata | 0.120031629*L^2.00 | Edwards et al. 2009 |
| Haliplus cf. obliquus | Coleoptera (larvae) | 0.002554241*L^2.76 | Burgherr & Meyer 1997 |
| Herpetocypris brevicaudata | Cadona | 0.108*(0.81058*L^2.4755) | Jimenez et al. 2011 |
| Herpetocypris helenae | Cadona | 0.108*(0.81058*L^2.4755) | Jimenez et al. 2011 |
| Herpetocypris reptans | Cadona | 0.108*(0.81058*L^2.4755) | Jimenez et al. 2011 |
| Hydracarina | Hydracarina | 0.132655465*L^1.66 | Baumgärtner & Rothhaupt 2003 |
| Hydroglyphus cf. geminus | Hydroporus spp. | 0.0618*L^2.502 | Smock 1980 |
| Chironomus spp. | Chironomus spp. | 0.00068*L^2.620 | Benke et al. 1999 |
| Chironomus tentans/pallidivittatus | Chironomus spp. | 0.00068*L^2.620 | Benke et al. 1999 |
| Ischnura elegans | Ischnura spp. | 0.0015*L^2.904 | Benke et al. 1999 |
| Isocypris beauchampi | Cadona | 0.108*(0.81058*L^2.4755) | Jimenez et al. 2011 |
| Kiefferulus spp. | Chironominae | 0.0059*L^2.099 | Benke et al. 1999 |
| Libelulla quadrimaculata | Libellula spp. | 0.0151*L^2.393 | Benke et al. 1999 |
| Macropelopia spp. | Tanypodinae | 0.0026*L^2.503 | Benke et al. 1999 |
| Micronecta spp. juv. | Sigara sp. | 0.0031*L^2.904 | Smock 1980 |
| Micropsectra spp. | Tanytarsini | 0.0008*L^2.728 | Benke et al. 1999 |
| Microtendipes pedellus gr. | Chironominae | 0.0059*L^2.099 | Benke et al. 1999 |
| Microtendipes spp. | Chironominae | 0.0059*L^2.099 | Benke et al. 1999 |
| Paratanytarsus austriacus | Taytarsus spp. | 0.0012*L^2.294 | Benke et al. 1999 |
| Paratanytarsus spp. | Taytarsus spp. | 0.0012*L^2.294 | Benke et al. 1999 |
| Platycnemis pennipes | Coenagrionidae | 0.0051*L^2.785 | Benke et al. 1999 |
| Polypedilum (Polypedilum) spp. | Polypedilum spp | 0.0010*L^2.761 | Benke et al. 1999 |
| Procladius (Holotanypus) spp. | Procladius spp. | 0.00077*L^2.693 | Benke et al. 1999 |
| Procladius (Psilotanypus) | Procladius spp. | 0.00077*L^2.693 | Benke et al. 1999 |
| Procladius (Psilotanypus) spp. | Procladius spp. | 0.00077*L^2.693 | Benke et al. 1999 |
| Psectrocladius sordidellus gr. | Psectrocladius barbimanus | (0.0652 + 0.0765*L)^3/(1-0.042) | Lindegaard & Jónasson (1979) + Benke et al. 1999 (AFDM conversion) |
| Sigara falleni | Sigara sp. | 0.0031*L^2.904 | Smock 1980 |
| Sigara striata | Sigara sp. | 0.0031*L^2.904 | Smock 1980 |
| Tanytarsus spp. | Taytarsus spp. | 0.0012*L^2.294 | Benke et al. 1999 |
| Zavrelimyia sp. | Tanypodinae | 0.0026*L^2.503 | Benke et al. 1999 |
| Dugesia gonocephala | Dugesia tigrina | 0.0089*L^2.145 | Benke et al. 1999 |

**Table S2**: Recorded invertebrate taxa and their species traits.

|  |  |  |  |  | **Locomotion** | | | | |
| --- | --- | --- | --- | --- | --- | --- | --- | --- | --- |
| **Taxon** | **Average biomass**  **(mg DM)** | **Life cycle**  **(yrs)** | **Body**  **flexibility** | **Body**  **form** | diving/swimming | burrowing | walking | (semi)sessile |  |
| **Turbellaria** |  |  |  |  |  |  |  |  |  |
| *Dugesia gonocephala* | 0.123 | ≤ 1 | >300° | Flattened |  |  | 10 |  |  |
| **Hirudinea** |  |  |  |  |  |  |  |  |  |
| *Erpobdella octoculata* | 20.707 | > 1 | >300° | Cylindrical | 3 |  | 7 |  |  |
| *Glossiphonia nebulosa* | 13.241 | ≤ 1 | >45-300° | Cylindrical |  |  | 10 |  |  |
| **Isopoda** |  |  |  |  |  |  |  |  |  |
| *Asellus* aquaticus | 0.013 | ≤ 1 | >10-45° | Flattened | 5 |  | 5 |  |  |
| **Ostracoda** |  |  |  |  |  |  |  |  |  |
| *Cypridopsis vidua* | 0.008 | ≤ 1 | <10° | Spherical | 10 |  |  |  |  |
| *Herpetocypris brevicaudata* | 0.347 | ≤ 1 | <10° | Spherical | 10 |  |  |  |  |
| *Herpetocypris helenae* | 0.610 | ≤ 1 | <10° | Spherical | 10 |  |  |  |  |
| *Herpetocypris reptans* | 0.733 | ≤ 1 | <10° | Spherical | 10 |  |  |  |  |
| *Isocypris beauchampi* | 0.190 | ≤ 1 | <10° | Spherical | 10 |  |  |  |  |
| **Hydracarina** |  |  |  |  |  |  |  |  |  |
| Hydracarina indet. | 0.119 | ≤ 1 | <10° | Spherical | 3 |  | 7 |  |  |
| **Ephemeroptera** |  |  |  |  |  |  |  |  |  |
| *Caenis horaria* | 0.006 | ≤ 1 | >10-45° | Flattened |  |  | 10 |  |  |
| *Cloeon dipterum* | 0.065 | ≤ 1 | >45-300° | Streamlined | 8 |  | 2 |  |  |
| *Ephemera danica* | 3.627 | > 1 | >45-300° | Cylindrical | 1 | 9 |  |  |  |
| **Odonata** |  |  |  |  |  |  |  |  |  |
| *Coenagrion puella* | 16.572 | ≤ 1 | >45-300° | Cylindrical | 3 |  | 7 |  |  |
| *Ischnura elegans* | 4.196 | ≤ 1 | >45-300° | Cylindrical | 4 |  | 6 |  |  |
| *Libelulla quadrimaculata* | 28.852 | > 1 | >10-45° | Flattened | 2 | 4 | 4 |  |  |
| *Platycnemis pennipes* | 5.534 | > 1 | >45-300° | Cylindrical | 4 |  | 6 |  |  |
| **Trichoptera** |  |  |  |  |  |  |  |  |  |
| *Athripsodes aterrimus* | 0.067 | ≤ 1 | <10° | Cylindrical |  | 5 | 5 |  |  |
| **Coleoptera** |  |  |  |  |  |  |  |  |  |
| *Haliplus* cf. *obliquus* | 1.082 | ≤ 1 | >10-45° | Streamlined | 5 |  | 5 |  |  |
| *Hydroglyphus* cf. *geminus* | 1.237 | ≤ 1 | >10-45° | Cylindrical | 10 |  |  |  |  |
| **Diptera** |  |  |  |  |  |  |  |  |  |
| *Ablabesmyia* cf. *phatta* | 0.048 | ≤ 1 | >300° | Cylindrical |  |  | 10 |  |  |
| *Cladopelma lateralis* gr. | 0.011 | ≤ 1 | >300° | Cylindrical |  |  | 10 |  |  |
| *Cladotanytarsus mancus* gr. | 0.007 | ≤ 1 | >300° | Cylindrical |  | 10 |  |  |  |
| Clinotanypus sp. | 0.157 | ≤ 1 | >300° | Cylindrical | 2 | 8 |  |  |  |
| *Corynoneura* *scutellata* gr. | 0.011 | ≤ 1 | >300° | Cylindrical |  | 6 | 4 |  |  |
| *Cryptochironomus* spp. | 0.027 | ≤ 1 | >300° | Cylindrical |  | 10 |  |  |  |
| *Demicryptochironomus* spp. | 0.140 | ≤ 1 | >300° | Cylindrical |  | 10 |  |  |  |
| *Dicrotendipes* spp. | 0.067 | ≤ 1 | >300° | Cylindrical |  | 10 |  |  |  |
| *Endochironomus* cf. *tendes* | 0.059 | ≤ 1 | >300° | Cylindrical |  | 10 |  |  |  |
| *Chironomus tentans/pallidivittatus* | 2.308 | ≤ 1 | >300° | Cylindrical |  | 10 |  |  |  |
| *Kiefferulus* spp. | 0.281 | ≤ 1 | >300° | Cylindrical |  | 10 |  |  |  |
| *Macropelopia* spp. | 0.051 | ≤ 1 | >300° | Cylindrical | 1 | 9 |  |  |  |
| *Micropsectra* spp. | 0.044 | ≤ 1 | >300° | Cylindrical |  | 10 |  |  |  |
| *Microtendipes pedellus* gr. | 0.271 | ≤ 1 | >300° | Cylindrical |  | 10 |  |  |  |
| *Paratanytarsus austriacus* | 0.009 | ≤ 1 | >300° | Cylindrical |  | 10 |  |  |  |
| *Polypedilum (Polypedilum)* spp. | 0.115 | ≤ 1 | >300° | Cylindrical |  |  | 5 | 5 |  |
| *Procladius (Holotanypus*) spp. | 0.060 | ≤ 1 | >300° | Cylindrical | 2 | 8 |  |  |  |
| *Procladius (Psilotanypus)* spp. | 0.019 | ≤ 1 | >300° | Cylindrical | 2 | 8 |  |  |  |
| *Psectrocladius sordidellus* gr. | 0.028 | ≤ 1 | >300° | Cylindrical |  | 10 |  |  |  |
| *Tanytarsus* spp. | 0.037 | ≤ 1 | >300° | Cylindrical |  | 10 |  |  |  |
| *Zavrelimyia* sp. | 0.037 | ≤ 1 | >300° | Cylindrical | 1 | 9 |  |  |  |
| Ceratopogonidae indet. | 0.121 | ≤ 1 | >300° | Cylindrical |  | 10 |  |  |  |
| **Heteroptera** |  |  |  |  |  |  |  |  |  |
| *Micronecta* spp. | 0.013 | ≤ 1 | <10° | Flattened | 7 |  | 3 |  |  |
| *Sigara falleni* | 0.930 | ≤ 1 | <10" | Flattened | 9 |  | 1 |  |  |
| *Sigara striata* | 0.801 | ≤ 1 | <10" | Flattened | 9 |  | 1 |  |  |

**References**

*Identification keys*

[Askew, R. R. 2004. The Dragonflies of Europe. 2nd edition. Harley Books, Colchester, UK.](http://paperpile.com/b/BWToip/gzlbx)

[Bauernfeind, E., and U. H. Humpesch. 2001. Die Eintagsfliegen Zentraleuropas (Insecta:Ephemeroptera): Bestimmung und Okologie [The Mayflies of Central Europe (Insecta: Ephemeroptera): Identification and Ecology. Vienna Natural History Museum, Vienna, Austria.](http://paperpile.com/b/BWToip/of6rk)

[Gonzalez Mozo, M. E., K. Martens, and A. Baltanás. 1996. A taxonomic revision of European Herpetocypris Brady and Norman, 1889 (Crustacea, Ostracoda). Bulletin van het Koninklijk Belgisch Institut voor Natuurwetenschappen Biologie 66:93–132.](http://paperpile.com/b/BWToip/DJk4D)

[Meisch, C. 2000. Freshwater Ostracoda of western and central Europe. Spektrum Akademischer Verlag, Heidelberg, Germany.](http://paperpile.com/b/BWToip/EnhwC)

[Rozkošný, R., editor. 1980. Klíč vodních larev hmyzu [Key to aquatic insect larvae]. Academia, Prague, Czech Republic.](http://paperpile.com/b/BWToip/Mn1vv)

[Tempelman, D., and T. van Haaren. 2009. Water- en Oppervlaktewantsen van Nederland [Aquatic and semiaquatic true bugs of Netherlands]. Jeugdbondsuitgeverij, Utrecht, Netherlands.](http://paperpile.com/b/BWToip/FohWj)

Waringer, J., and W. Graf. 2011. Atlas der mitteleuropäischen Köcherfliegenlarven [Atlas of Central European Trichoptera Larvae]. Erik Mauch Verlag, Dinkelscherben, Germany.

[Wiederholm, T. 1983. Chironomidae of the holarctic region. Keys and diagnoses. Part 1: larvae. Entomological Society of Lund, Lund, Sweden.](http://paperpile.com/b/BWToip/gG5B5)

*Length weight relationships*

Anderson, D. H., S. Darring, and A. C. Benke. 1998. Growth of crustacean meiofauna in a forested floodplain swamp: implications for biomass turnover. Journal of the North American Benthological Society 17:21–36.

Baumgärtner, D., and K. O. Rothhaupt. 2003. Predictive length–dry mass regressions for freshwater invertebrates in a pre‐alpine lake littoral. International Review of Hydrobiology 88:453–463.

Benke, A. C., A. D. Huryn, L. A. Smock, and J. B. Wallace. 1999. Length-mass relationships for freshwater macroinvertebrates in North America with particular reference to the southeastern United States. Journal of the North American Benthological Society 18:308–343.

Burgherr, P., and E. I. Meyer. 1997. Regression analysis of linear body dimensions vs. dry mass in stream macroinvertebrates. Archiv für Hydrobiologie 139:101–112.

Jimenez, A., M. D. Rennie, W. G. Sprules, and J. La Rose. 2011. Temporal changes in the benthic invertebrate community of Lake Simcoe, 1983–2008. Journal of Great Lakes Research 37:103–112.

Lindegaard, C., and P. M. Jónasson. 1979. Abundance, population dynamics and production of zoobenthos in Lake Mývatn, Iceland. Oikos 32:202–227.

Nolte, U. 1990. Chironomid biomass determination from larval shape. Freshwater Biology 24:443–451.

Pavlov, A. M., and I. M. Zubina. 1990. Relationship of weight to body dimensions in dragonfly larvae. Hydrobiological Journal 26:99–103.

Smock, L. A. 1980. Relationships between body size and biomass of aquatic insects. Freshwater Biology 10:375–383.

Tokeshi, M. 1985. Life-cycle and production of the burrowing mayfly, Ephemera danica: a new method for estimating degree-days required for growth. Journal of Animal Ecology 54:919–930.

*Traits*

Schmidt-Kloiber, A., and D. Hering. 2015. www.freshwaterecology.info – An online tool that unifies, standardises and codifies more than 20,000 European freshwater organisms and their ecological preferences. Ecological Indicators 53:271–282.

[Šporka, F. 2003. Aquatic macroinvertebrates of Slovakia: species list and autecological characteristics. Slovenský hydrometeorologický ústav, Bratislava, Slovakia.](http://paperpile.com/b/BWToip/C3SFM)

[Tachet, H., P. Richoux, M. Bournaud, and P. Usseglio-Polatera. 2000. Invertébrés d’eau douce: systématique, biologie, écologie [Freshwater invertebrates: systematics, biology, ecology]. CNRS éditions, Paris, France.](http://paperpile.com/b/BWToip/feD8U)

**C. Supplementary results**

**Table S3:** Fish performance effects, evaluated using linear mixed models. Survival was evaluated using a binomial glmm with Wald chi-squared tests; length and condition were evaluated using gaussian lmms with F tests and Kenward-Roger df estimation. Effects with p≤0.05 are bolded to indicate significance. Interactions involving ecotype were not significant (p>0.18) so those effects are omitted for brevity. Semi-partial r^2^ values indicate the relative explanatory power of fixed effects. R^2^m and R^2^c refer to marginal and conditional R^2^, which quantify the approximate variation in the response explained by fixed effects and fixed plus random effects, respectively.

| **Response** | **Group** | **Fixed Effect** | **stat** | **df** | **p** | **partial r^2^** | **R^2^m** | **Random Effect** | **p** | **R^2^c** |
| --- | --- | --- | --- | --- | --- | --- | --- | --- | --- | --- |
| Survival rate | Ecotypes | Macrophytes | 0.03 | 1 | 0.869 | 0 | 0.62 | Block | 0.123 | 0.94 |
|  |  | **Fish Density** | 18.15 | 1 | **<0.001** | 0.115 |  | Tank | **<0.001** |  |
|  |  | M x F | 0.42 | 1 | 0.426 | 0 |  |  |  |  |
|  |  | **Ecotype** | 20.41 | 1 | **<0.001** | 0.037 |  |  |  |  |
|  | F2s | **Macrophytes** | 16.39 | 1 | **<0.001** | 0.112 | 0.91 | Block | **0.008** | 0.97 |
|  |  | **Fish Density** | 32.89 | 1 | **<0.001** | 0.648 |  |  |  |  |
|  |  | M x F | 1.93 | 1 | 0.165 | 0.118 |  |  |  |  |
| Length | Ecotypes | Macrophytes | 0.03 | 1,12 | 0.865 | 0 | 0.16 | Block | 0.135 | 0.37 |
|  |  | **Fish Density** | 23.18 | 1,12 | **<0.001** | 0.033 |  | Tank | **<0.001** |  |
|  |  | M x F | 0.11 | 1,12 | 0.743 | 0.001 |  |  |  |  |
|  |  | Ecotype | 2.54 | 1,726 | 0.111 | 0.001 |  |  |  |  |
|  | F2s | Macrophytes | 2.10 | 1,12 | 0.173 | 0.019 | 0.20 | Block | 0.074 | 0.36 |
|  |  | **Fish Density** | 37.42 | 1,12 | **<0.001** | 0.124 |  | Tank | **<0.001** |  |
|  |  | M x F | 0.57 | 1,12 | 0.464 | 0 |  |  |  |  |
| Condition (log) | Ecotypes | **Macrophytes** | 4.58 | 1,12 | **0.054** | 0.016 | 0.13 | Block | 1 | 0.22 |
|  |  | **Fish Density** | 20.47 | 1,12 | **<0.001** | 0.027 |  | Tank | **<0.001** |  |
|  |  | M x F | 0.05 | 1,12 | 0.821 | 0.001 |  |  |  |  |
|  |  | **Ecotype** | 5.53 | 1,727 | **0.019** | 0.003 |  |  |  |  |
|  | F2s | Macrophytes | 3.95 | 1,10 | 0.074 | 0.015 | 0.09 | Block | **0.040** | 0.19 |
|  |  | **Fish Density** | 25.71 | 1,12 | **<0.001** | 0.077 |  | Tank | **0.002** |  |
|  |  | **M x F** | 4.81 | 1,12 | **0.049** | 0.016 |  |  |  |  |

**Table S4:** Summary of marker density for QTL analysis.

| **Chromosome** | **# Markers** | **Length** | **Avg. spacing** | **Max spacing** |
| --- | --- | --- | --- | --- |
| **1** | 26 | 69.2 | 2.8 | 19.3 |
| **2** | 25 | 105.6 | 4.4 | 24.0 |
| **3** | 19 | 43.8 | 2.4 | 31.0 |
| **4** | 28 | 130.8 | 4.8 | 25.4 |
| **5** | 15 | 66.1 | 4.7 | 21.3 |
| **6** | 22 | 72.9 | 3.5 | 24.7 |
| **7** | 57 | 153.1 | 2.7 | 43.9 |
| **8** | 38 | 38.8 | 1.0 | 7.9 |
| **9** | 39 | 117.3 | 3.1 | 45.0 |
| **10** | 8 | 86.3 | 12.3 | 50.9 |
| **11a** | 14 | 41.3 | 3.2 | 29.3 |
| **11b** | 11 | 4.9 | 0.5 | 1.3 |
| **12** | 17 | 62.6 | 3.9 | 28.6 |
| **13** | 19 | 115.7 | 6.4 | 48.1 |
| **14** | 15 | 85.3 | 6.1 | 40.8 |
| **15** | 25 | 80.3 | 3.3 | 16.0 |
| **16** | 6 | 69.1 | 13.8 | 56.3 |
| **17** | 30 | 47.3 | 1.6 | 14.7 |
| **18** | 9 | 22.2 | 2.8 | 7.1 |
| **19** | 6 | 70.9 | 14.2 | 34.7 |
| **20** | 15 | 43.6 | 3.1 | 26.7 |
| **21** | 10 | 50.3 | 5.6 | 18.9 |
| **Overall** | 454 | 1577.5 | 3.7 | 56.3 |

**Table S5:** Summary of QTL analyses on growth (inferred from final size) and size-corrected mouth morphological traits. P-values for identification of loci with significant LOD scores are based on 1000 permutations with an alpha of 0.05. Percent variance explained is from an ANOVA of significant QTL and density effects. Highest genotype indicates whether the trait is maximized in the homozygous stream (SS) or homozygous lake (LL) genotype. For loci with interactive effects with density, we give the genotype with the highest value in the low fish density treatment, where the mean and variation in growth were much higher, increasing the potential for QTL detection.

|  |  | **p-value** | | **% variance explained** | | |  |
| --- | --- | --- | --- | --- | --- | --- | --- |
|  |  | **Main effect** | **x Density effect** | **Main effect** | **x Density effect** | **Density effect** | **Highest genotype** |
| **Trait** | **Marker** |  |  |  |  |  |  |
| Final length (mm, log) | chrXVI_1268216 | 0.002 |  | 2.6 |  | 20 | SS |
|  | chrX_541801 |  | 0.018 |  | 1.4 |  | SS in Low |
|  | chrXII_6416748 |  | 0.035 |  | 1.6 |  | SS in Low |
| Final weight (mg, log) | chrXVI_1268216 | 0.014 |  | 1.9 |  | 25.8 | SS |
|  | chrX_541801 |  | 0.031 |  | 1 |  | SS in Low |
|  | chrXII_6416748 |  | 0.006 |  | 1.6 |  | SS in Low |
| Jaw protrusion | chrI_18936520 | 0.035 |  | 3 |  | 1.7 | LL |
| Gape | chrV_14013034 | 0.046 |  | 2.9 |  | 1.5 | SS |
|  | chrXIX_2927175 | 0.018 |  | 3.3 |  |  | LL |
| Buccal length | chrXX_18624458 | 0.016 |  | 3.1 |  | 0.8 | SS |

**Figure S2:** At higher density, which decreased total survival, the stream ecotype consistently survived better than the lake ecotype. Data are from the 20 mesocosms originally stocked with equal proportions of lake and stream ecotypes, at either high or low density. Symbol color indicates macrophyte presence and size indicates fish density.

**Figure S3:** Genetic composition of experimental fish based on 4000 autosomal RAD loci. a) PCA of wild-caught fish bred in the lab to supply mesocosms, including the two F0 parents of the F2 cross (male from lake and female from stream) and the 20 lake and 20 stream parents of the ecotypes. b) PCA of the same F0 parents and the F2s surviving in the mesocosms, colored by the five different F1 family groups that supplied the five experimental blocks. When reduced to only loci that were alternative homozygotes in the F0s, making F1s identical, the block effects disappear.

**Figure S4:** Linkage map for QTL analysis.

**Figure S5:** Phenotype distributions for juvenile ecotypes and F2s surviving the mesocosm experiment. All traits are size corrected (sc). Traits with significant (or marginally significant) differentiation between ecotypes when accounting for random effects of mesocosm and block are annotated.
